# Chondroid syringoma of the nose: A rare case report and literature review

**DOI:** 10.1016/j.ijscr.2024.109248

**Published:** 2024-01-11

**Authors:** Lulwah AlSaidan, Mariam Sarkhouh, Athary Alenezi, Humoud AlSabah, Ibrahim Al Aradi, Abdulmohsen Alterki

**Affiliations:** a Department of Otolaryngology, Head and Neck Surgery, Zain Hospital, Shuwaikh Medical Area, Kuwait; b Department of Otolaryngology, Head and Neck Surgery, Zain Hospital, Shuwaikh Medical Area, Kuwait; c Department of Dermatology, Asaad Al-Hamad Hospital, Shuwaikh Medical Area, Kuwait

**Keywords:** Chondroid syringoma, Mixed tumor of the skin, Rare

## Abstract

**Introduction and importance:**

A chondroid syringoma is an exceptionally rare benign lesion of the sweat glands also known as mixed tumor of the skin (MTS). It can occur in different areas of the head and neck such as the lips, cheek, nose and scalp (Gotoh et al., 2022 [1]). It is usually painless and grows slowly. Based on pathological features it can be differentiated into apocrine or eccrine (*Mixed cutaneous tumor: chondroid syringoma a case report*, 2019 [2]).

**Case presentation:**

Our patient presented with a nasal lesion in the left soft triangle, progressively increasing in size. He did not undergo any surgeries to the nose or any history of trauma. Due to the COVID-19 lockdown our patient did not seek early medical advice. In addition, the implementation of facemasks enabled for the concealment of the abnormality, which reduced the need of seeking treatment.

**Clinical discussion:**

Chondroid syringoma is a non-ulcerative tumor that grows slowly with an average diameter between 0.5 and 3 cm, however lesions reaching 9 cm have been also seen (Wan et al., 2018 [4]). The mainstay method of management is surgical excision while maintaining the aesthetic appearance of the patient.

**Conclusion:**

Owing to its rarity, clinical misdiagnosis is common, however absolute diagnosis is achieved by histopathology. This case delineates the rarity of this lesion and the mainstay method of management, which is surgical excision.

## Highlights

• A chondroid syringoma is a very rare benign tumor of the sweat glands also known as mixed tumor of the skin (MTS).
• It’s considered extremely rare, and according to literature the incidence is really low ranging between 0.01%-0.098% with a male predominance
• Definite diagnosis is done by histopathology results of the excised mass
• The mainstay method of management is surgical excision while maintaining the aesthetic appearance of the patient.

## 1 Introduction

Chondroid syringoma is considered an uncommon tumor that is benign in nature. Because of how rare the condition is, it can be misdiagnosed as dermal nevi, cysts, or other cutaneous adnexal tumors. It is more common among males aging 20 to 60 years [3]. It usually occurs in the lips, cheek, nose, and scalp [1]. Histopathology plays an important role in diagnosing this tumor. MTS has some common characteristics with pleomorphic adenoma histologically [4]. Our patient's main complaint was aesthetic because of the central presentation of the mass. An excisional biopsy is the gold standard management, while making sure the normal aesthetic anatomy is maintained. Chondroid syringoma is an important differential diagnosis for cutaneous masses although rare; otorhinolayringologists should consider it in their differential diagnosis.

This work has been reported in line with the SCARE 2023 criteria [5].

## 2 Case presentation

A 35 years old gentleman previously healthy, presented to the ENT clinic in Kuwait with 7 months history of a nasal lesion on the left soft triangle. The lesion started as a small soft oval shaped mass and increased in size gradually around 3 mm per month. It was not associated with pain, fever, bleeding, or difficulty breathing. There was no history of trauma or any surgeries to the nose. His family history was clear from similar lesions.

**Fig. 1:**
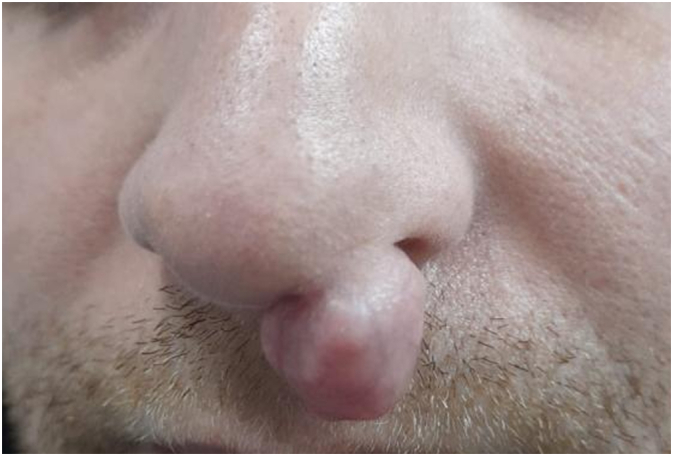
Pre-operative picture of our patient's chondroid syringoma.

On examination, he was vitally stable and afebrile. A pedunculated left soft triangle nodule was noted on the nose, measuring around 2 × 1.5 cm. Its oval in shape and the color is similar to the patient's skin. On palpation, it was mobile with a peduncle attached to the soft triangle. It was non-tender and had normal temperature. There were no palpable lymph nodes or similar lesions on his body. Full ENT exam was done that was unremarkable. (Fig. 1).

A biopsy was taken which showed a well-circumscribed biphasic tumor with admixed epithelial and myoepithelial/mesenchymal components. Epithelial component composed of branching glands lined by two layer of epithelial cells or small duct. Mesenchymal component shows a myxoid stroma, and clusters of spindle to epithelioid myoepithelial cells. Diagnosed as chondroid syringoma (mixed tumor of the skin).

**Fig. 2:**
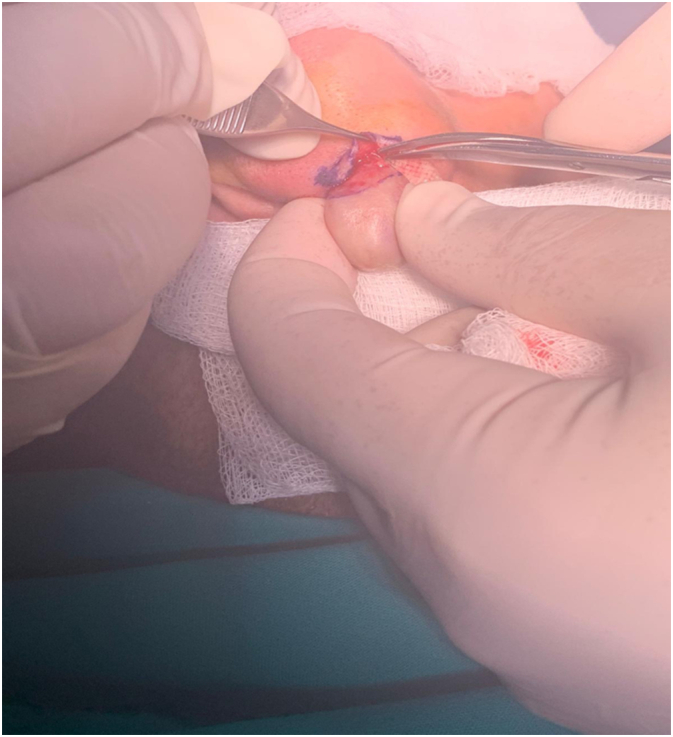
Intraoperative flap elevation and dissection of the tumor.

**Fig. 3:**
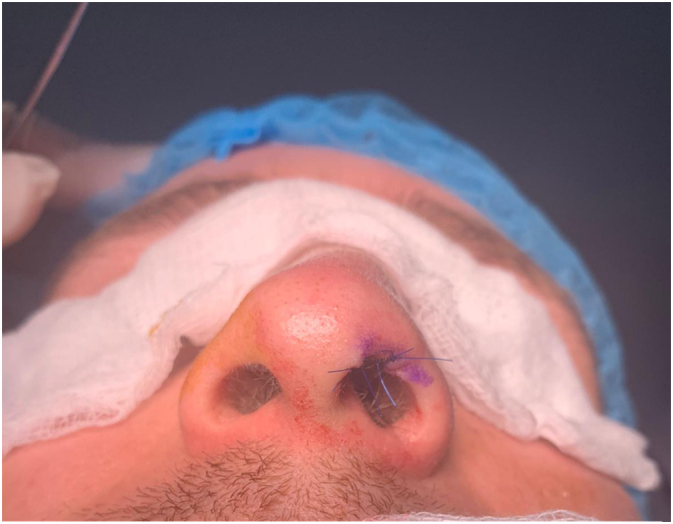
Immediately post-operative after excision.

**Fig. 4:**
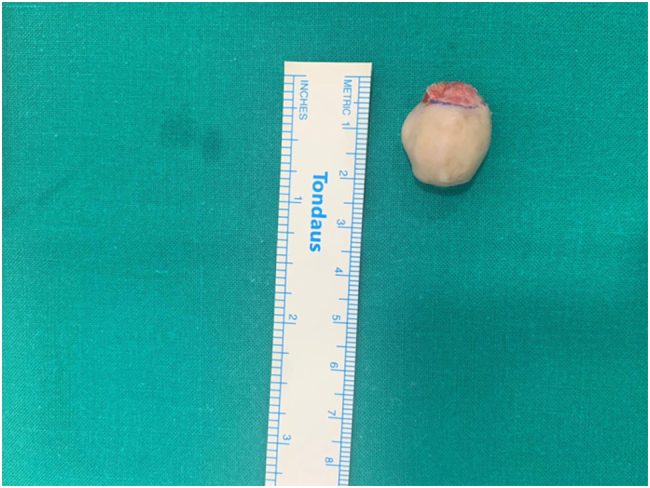
Excised chondroid syringoma measuring around 2 × 2 cm.

Patient was scheduled for a surgical excision and core fillet flap, that was done under local anesthesia. The incision was made in a flap from the anterior skin portion overlying the tumor. (Fig. 2) Next the tumor was excised from anterior and posterior aspects of the tumor base near the nostril. The flap was folded on the tip of the nostril and secured by interrupted square matrices as seen in the pictures. Follow up was done showing great wound healing and no complications. (Fig. 3, Fig. 4).

**Fig. 5:**
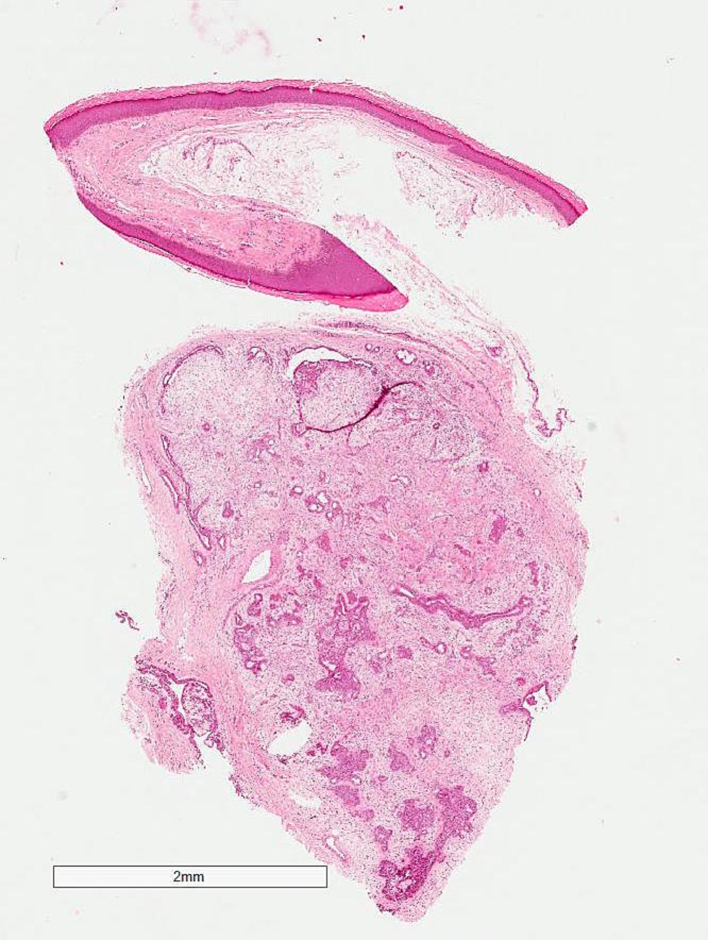
Lower power: well-circumscribed biphasic tumor with admixed epithelial and myoepithelial/mesenchymal components. Epithelial component composed of branching glands lined by two layers of epithelial cells or small ducts. Mesenchymal component shows a myxoid stroma and clusters of spindle to epithelioid myoepithelial cells.

**Fig. 6:**
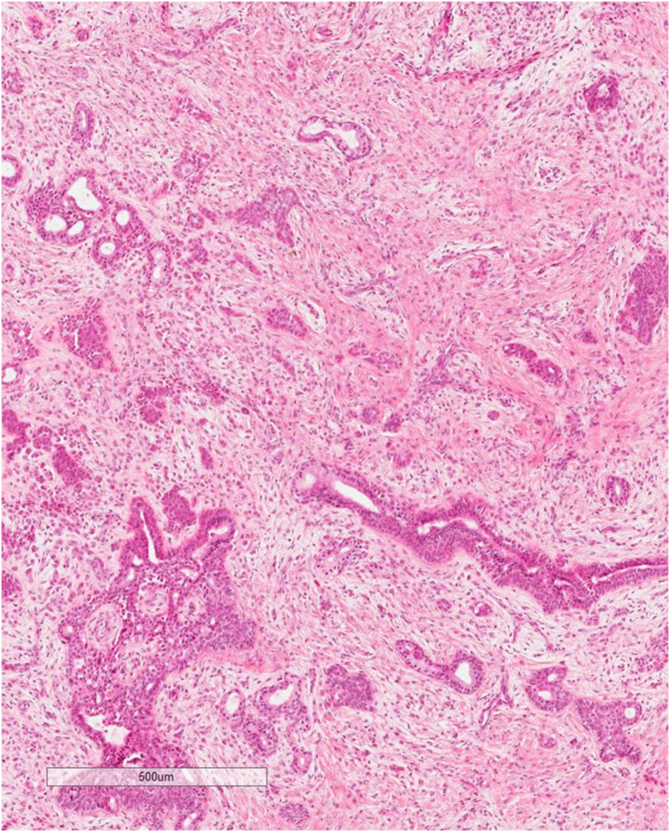
Mesenchymal component shows a myxoid stroma and clusters of spindle to epithelioid myoepithelial cells.

**Fig. 7:**
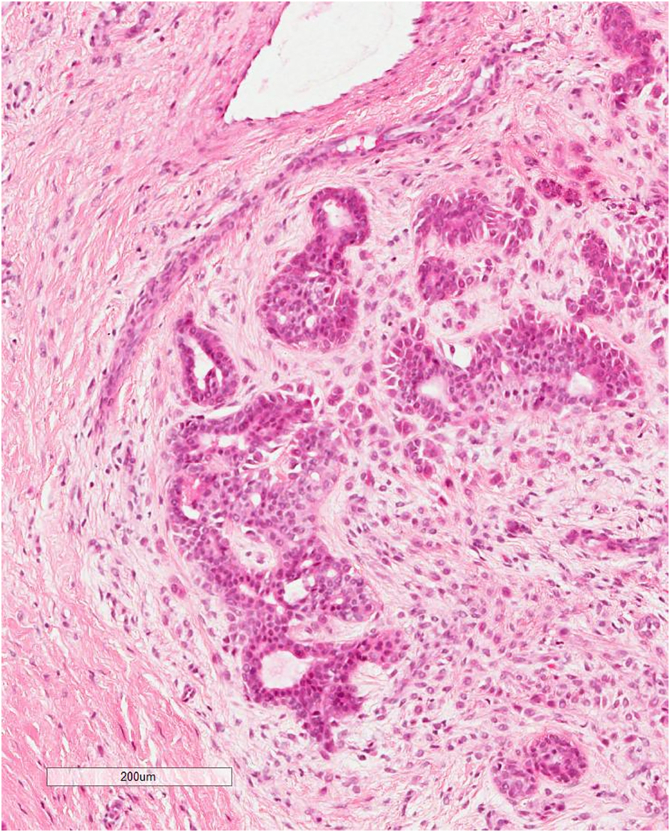
High power: Epithelial component composed of branching glands lined by two layers of epithelial cells or small ducts.

The excisional biopsy showed a well-circumscribed mass filling whole dermis is formed of alveolar branched tubular ducts lined with cuboidal cells and filled with sweats and mixed with abundant myxoid stroma of fibroblasts. The overlying epidermis is atrophied, confirming the diagnosis. (Fig. 5, Fig. 6, Fig. 7).

## 3 Discussion

Mixed tumors of the skin (MTS), also known as chondroid syringomas contain mesenchymal and epithelial contents. It's considered extremely rare, and according to literature the incidence is really low ranging between 0.01 %–0.098 % with a male predominance of ages between 20 and 40 years, mostly appearing on the head and neck [1,3]. A chondroid syringoma was given the name by Hirsch and Helwig due to the presence of sweat glands embedded in cartilaginous stroma. Based on pathological features it can be differentiated into apocrine or eccrine [2]. It usually presents as a single subcutaneous nodule with a smooth surface. It can be skin colored or light red and is firm or hard on palpation giving account to its interstitial content. Chondroid syringoma is a non-ulcerative tumor that grows slowly with an average diameter between 0.5 and 3 cm, however lesions reaching 9 cm have been also seen [4].

Definite diagnosis is done by histopathology results of the excised mass. Five histological presentations were suggested by Hirsch and Helwig which are: groups of cuboidal or polygonal cells, ducts containing of one or two rows of cuboidal cells, some keratinous cysts, intercommunicating tubuloalveolar complexes lined with two or more rows of cuboidal cells, and a matrix. They can have all five features or only some of them [6].

The mainstay method of management is surgical excision while maintaining the aesthetic appearance of the patient. It has a very low possibility of recurrence post-operatively. However, malignant MTS also exists and has particular characteristics under microscopy which are: satellite lesions, tumor necrosis, increase fission of nuclei, heterogeneous enlargement of cells, and infiltrative growth of the margin. Nevertheless, recurrence of malignant MTS is also rare after excision but still requires proper follow up [4]. Benign MTS usually presents in the head and neck region in 80 % of cases, yet malignant MTS most commonly occur on the extremities and trunk [7,8]**.** .Malignant MTS usually appears in woman more than men with a size more than 3 cm [8]. In very rare cases, chondriod syringoma that is left untreated for a long time can transform to malignant MTS with metastasis [9].

## 4 Conclusion

Chondroid syringoma, also known as mixed tumor of the skin is considered a rare benign tumor that most commonly presents in the head and neck region, making it an important differential diagnosis to consider among otorhinolayrngologists. Definite diagnosis is done via histopathology and treatment is by surgical excision.

## Consent

Written informed consent was obtained from the patient for publication of this case report and accompanying images. A copy of the written consent is available for review by the Editor-in-Chief of this journal on request.

## Ethical approval

Ethical approval was waived by the authors' institution.

## Funding

This research did not receive any specific grant from funding agencies in the public, commercial, or not-for-profit sectors.

## Author contribution

Dr Lulwah AlSaidan; data collection, data analysis, and write up.

Dr Mariam Sarkhouh; data collection and data analysis

Dr Athary Alenezi; data collection

Dr Humoud Al-Sabah; data analysis and contribution.

Dr Ibrahim Al Aradi; data analysis and contribution.

Dr Abdulmohsen AlTerki; data analysis and contribution.

## Guarantor

Dr Lulwah AlSaidan

## Research registration number

Name of the registry: Not applicable

2. Unique identifying number or registration ID: Not applicable

3. Hyperlink to your specific registration (must be publicly accessible and will be checked): not applicable

## Declaration of competing interest

There is no conflict of interest to declare by any of the authors of this study.
